# Cross-lagged analysis of interdependence and psychological symptom clusters in postpartum women: A prospective study

**DOI:** 10.1097/MD.0000000000045709

**Published:** 2025-10-31

**Authors:** Yunjuan Ji, Lili Xue, Liping Chen

**Affiliations:** a Department of Obstetrics, The First People’s Hospital of Nantong, Nantong City, Jiangsu Province, China.

**Keywords:** cross-lagged panel analysis, depression, interdependence relationship, postpartum, post-traumatic stress disorder, psychological resilience

## Abstract

The aim of this study is to investigate the longitudinal interaction mechanism between interdependence and clusters of psychological emotional symptoms in postpartum women. Three waves of follow-up assessments were conducted on 389 postpartum women at T1 (before discharge), T2 (42 days post-delivery), and T3 (3 months post-delivery). The assessments utilized the general information questionnaire, the 10-item Connor–Davidson resilience scale (CD-RISC-10), the Edinburgh Postnatal depression scale (EPDS), the post-traumatic stress disorder (PTSD) Checklist-Civilian version (PCL-C-7), and the mutuality scale (MS) to evaluate resilience, depression, PTSD, and marital mutuality. Cross-lagged panel analysis was employed to examine the causal paths between variables over time. The autoregressive effect indicated that all variables exhibited significant temporal stability (β = 0.50–0.65, *P* < .001), with resilience demonstrating the highest stability (T1 → T2: β = 0.65; T2 → T3: β = 0.62); The protective pathway revealed that resilience significantly enhanced the subsequent marital relationship (T1 → T2: β = 0.28; T2 → T3: β = 0.26), and a negative marital relationship predicted depressive symptoms (T2 → T3: β = −0.22). Resilience also directly reduced the risk of depression (T2 → T3: β = −0.23); and Inter-symptom reinforcement showed that depression increased the risk of subsequent PTSD (T1 → T2: β = 0.38; T2 → T3: β = 0.42), and PTSD exacerbation was followed by depression (T2 → T3: β = 0.37). Psychological resilience indirectly ameliorates emotional symptoms by bolstering marital relationships, while depression and PTSD establish a bidirectional vicious cycle. The research endorses a three-tiered prevention strategy that focuses on fostering resilience and intervening in partner relationships during the early postpartum period.

## 1. Introduction

Childbirth, as a significant life event, marks the beginning of a period of intense physical and mental changes for women as they adapt to the postpartum stage. Numerous studies have shown that the postpartum period is a high-risk window for psychological disorders.^[1,2]^ In the early postpartum period, new mothers face multiple physical recovery challenges, such as wound pain, difficulty in lactation, and sleep deprivation. Simultaneously, they are under tremendous psychological pressure, including concerns about the newborn’s health (such as jaundice and feeding issues), fear of improper parenting, doubts about their own competence, and uncertainty about body changes and the shift in social role focus.^[3–5]^ These complex physical and mental changes and challenges not only constitute stressors themselves but also significantly increase the risk of negative psychological and emotional symptoms in new mothers. Among these, postpartum depression (PPD) and postpartum post-traumatic stress disorder (PP-PTSD) are the most prominent representatives of emotional disorders, with rates as high as 39.96%^[4]^ and 18.1%,^[6]^ respectively. These disorders often coexist, forming a “psychological and emotional symptom cluster” characterized by low mood, anxiety, intrusive memories, avoidance behaviors, and hypervigilance,^[7,8]^ which not only severely impairs the quality of life and parenting ability of new mothers but also has long-term negative impacts on the mother-infant bond and the early development of children.

This study integrates psychological stress theory,^[9]^ Conservation of Resources (COR) theory,^[10]^ and the family stress model^[11]^ to construct a comprehensive theoretical framework explaining protective mechanisms for postpartum mental health. Psychological stress theory emphasizes individuals’ cognitive appraisal and coping processes in response to environmental demands; COR theory posits that individuals strive to obtain, protect, and maintain valued resources, with resource loss leading to psychological stress; the family stress model focuses on how family systems cope with stress and adapt to change. Together, these frameworks indicate that postpartum mental health depends on a dynamic balance between internal resources, external relational resources, and stressors. Within this framework, psychological resilience represents the core internal resource for coping with stress and adaptation.^[12]^ Higher resilience enables postpartum women to more effectively manage postpartum pressures, reducing depression and PTSD risks. Dyadic coping serves as a key external relational resource, extending beyond general marital satisfaction to emphasize partners’ quality of emotional sharing, mutual support, value resonance, and deep emotional connection.^[13]^ During the postpartum period, this profound emotional connection provides essential emotional support and practical assistance.

In this study, “dyadic coping” specifically refers to the quality of couple interactions across 3 dimensions: emotional sharing, mutual support behaviors, and shared meaning-making within the postpartum context. Compared to general marital satisfaction, it places greater emphasis on relationship reciprocity and emotional depth; compared to social support, it focuses more on emotional resonance and shared values within intimate relationships. This conceptual choice reflects the unique nature of the postpartum period, where mothers require deeper emotional connections and value affirmation beyond superficial satisfaction or instrumental support. Theoretically, psychological resilience likely influences dyadic coping through^[14,15]^: cognitive-affective pathways: highly resilient individuals tend toward positive interpretations of partner behaviors, reducing negative attributions; behavioral interaction pathways: resilience promotes constructive communication and emotional expression, enhancing reciprocal interactions; and stress-buffering pathways: resilience reduces spillover of personal stress into relationships, protecting relationship quality. Conversely, high-quality dyadic coping may foster psychological resilience by providing a secure base, enhancing self-efficacy, and facilitating positive emotional sharing. Although existing studies have separately examined psychological resilience, marital relationships, and postpartum mental health, 3 significant gaps remain: First, research integrating internal resources (psychological resilience), relational resources (dyadic coping), and psychological symptom clusters within a unified dynamic framework is lacking. Second, most studies employ cross-sectional designs, limiting clarification of directional and temporal relationships between variables. Third, understanding remains limited regarding how protective resources mutually influence each other and collectively address stress challenges.

Based on this theoretical framework and existing gaps, we propose:

H1: Levels of postpartum psychological resilience, dyadic coping, and psychological symptom clusters will demonstrate significant temporal trends during postpartum period (pre-discharge to 3 months postpartum);

H2: Prior psychological resilience will significantly predict subsequent dyadic coping quality, with this relationship stronger than the reverse pathway;

H3: Psychological resilience and dyadic coping will form a protective resource system demonstrating synergistic protective effects against psychological symptom clusters;

H4: Psychological symptom clusters will significantly erode psychological resilience resources and impair dyadic coping quality, forming a vicious cycle. Regarding analytical methods, we thoroughly considered the choice between traditional cross-lagged panel modeling (CLPM) and random intercept cross-lagged panel modeling (RI-CLPM). Although RI-CLPM distinguishes between-person from within-person effects, this study focuses on dynamic predictive relationships between variables rather than purely within-person change, with relatively limited sample size. Thus, traditional CLPM was selected. We ensured model robustness through: testing variable stationarity; comparing model fit across lag structures; using full information maximum likelihood estimation for missing data; and conducting serial autocorrelation and cross-correlation tests.

By integrating multiple theoretical frameworks and employing rigorous analytical methods, this study aims to elucidate protective mechanisms for postpartum mental health, providing scientific evidence for developing precise prevention and intervention strategies. Given this, the present study, which employs the cross-lagged panel analysis research method, conducted a follow-up investigation of postpartum women at 3 key time points: before discharge (T1), 42 days postpartum (T2), and 3 months postpartum (T3). The primary objective is to explore the stability of 2 key factors, psychological resilience and marital interdependence, during the postpartum period and their dynamic mutual influence. The focus is on revealing the direction, intensity, and dynamic evolution trajectory of the mutual prediction among psychological resilience, marital interdependence, and the cluster of psychological and emotional symptoms at the 3 time points. This study aims to fill the research gap on the dynamic interaction between protective factors and risk symptom clusters in the postpartum period, and to provide a deeper understanding of the protective mechanisms of postpartum mental health and develop precise early prevention and timely intervention strategies.

## 2. Research subjects and methods

### 2.1. Research subjects

The women selected as research subjects prospectively were those who delivered at the Obstetrics Department of Nantong First People’s Hospital between January 2025 and May 2025 and consented to participate in the follow-up study post-delivery. Inclusion criteria were as follows: hospitalization in the obstetric department of our hospital and completion of delivery; singleton live birth with a gestational age of at least 37 weeks and a birth weight of at least 2500 g; intention to undergo the routine 42-day postpartum checkup at our hospital and commitment to completing 3 questionnaire assessments; and the mother’s understanding of the research purpose and voluntary signing of the informed consent form. Exclusion criteria included: newborn transferred to the NICU for 7 days or more post-birth, or diagnosed with severe congenital malformations or genetic metabolic diseases; severe complications during delivery or postpartum, such as eclampsia or postpartum hemorrhage; Previously or currently diagnosed with mental disorders; and Unmarried or without a stable partner. The sample size was calculated based on the core principles of the cross-lagged panel model, which can stably estimate all free parameters of the model (a total of 34 free parameters). The Monte Carlo simulation function of Mplus in R was utilized, with the minimum target effect size of the cross-lagged path β = 0.20, α = 0.05 two-tailed test, and the target power ≥ 0.80. The simulation accounted for the autoregressive coefficient (0.6), measurement error (loading 0.7), and 15% random missing data. An additional 20% dropout rate was factored in, and the initial recruitment target was set at 400 cases. Ultimately, 389 patients completed the 3 follow-ups. This study was approved by the Ethics Committee of Nantong First People’s Hospital, with the approval number (2025KT153).

### 2.2. Survey tools

#### 2.2.1. Psychological resilience assessment tools

The 10-item Connor-Davidson Resilience Scale (CD-RISC-10) was utilized in this study. The scale, which is unidimensional, was translated into Chinese by Wang et al.^[16]^ It comprises 10 items designed to evaluate an individual’s psychological resilience. Responses were measured on a 5-point Likert scale ranging from 0 (“never”) to 4 (“almost always”), yielding a total possible score from 0 to 40. A higher score signifies greater psychological resilience. The Cronbach α coefficient in this study was 0.896.

#### 2.2.2. Postpartum depression screening

The Edinburgh postnatal depression scale (EPDS)^[17]^ comprises 10 items, each scored on a 4-point scale reflecting symptom frequency (0 = “never” to 3 = “always”), resulting in a total score that ranges from 0 to 30. A score of 10 or higher indicates the presence of depressive symptoms. In this study, Cronbach α was 0.903.

#### 2.2.3. Postpartum post-traumatic stress disorder screening

Postpartum PTSD was screened using the Civilian Version of the PTSD Checklist (PCL-C-7).^[18]^ The scale comprises 7 items, each rated on a 1 to 5 scale based on the severity of symptoms (1 = “none” to 5 = “extremely severe”). The diagnostic criteria require that all of the following be met: A single item score of ≥3; At least one positive item in the reeexperiencing symptom cluster; At least 3 positive items in both the avoidance and hyperarousal symptom clusters; and A total score of ≥38. The scale exhibits good reliability and validity, with Cronbach α for each symptom ranging from 0.916 to 0.921, and the total scale Cronbach α being 0.910.

#### 2.2.4. Evaluation of the interdependence of marital relationship

The Chinese version of the Mutuality Scale (MS)^[19]^ was utilized. This scale comprises 4 dimensions and fifteen items (sharing joy, reciprocity, common values, love, and affection), employing a 5-point Likert scale (0 = “none” to 4 = “very much”). The total score spans from 0 to 60. The scoring rule involves calculating the average score of the items (total score divided by 15). Higher scores indicate a better quality of the relationship; an average score below 2.5 suggests a poor interdependent relationship. The Cronbach α for the Chinese version of the scale is 0.91.

### 2.3. Data collection methods

Upon the parturient’s admission to the hospital for childbirth, the researcher thoroughly explained the study’s objectives, procedures, and privacy safeguards. Only those who consented by signing the informed consent form were enrolled in the study. The study involved 3 follow-up surveys. The initial survey took place 24 to 48 hours prior to discharge (T1), during which a package containing CD-RISC-10, EPDS, PCL-C-7, and MS questionnaires was distributed on paper in the ward. The second survey occurred 42 days postpartum, with a tolerance of ±3 days (T2), where participants completed the questionnaires using electronic terminals, coinciding with the postpartum review outpatient clinic at our hospital. The third survey was conducted 3 months postpartum, with a tolerance of ±7 days (T3), through the distribution of a WeChat questionnaire. The study employed the following strategies for follow-up management. Initially, a dual contact system was established, linking the parturient with an emergency contact person. Three days before the third follow-up, a reminder notice was sent via WeChat. All questionnaires were programmed with pertinent logical skip questions.

### 2.4. Statistical analysis

Data analysis was conducted using SPSS 26.0 (IBM Corporation, Armonk) and Mplus 8.2 (Muthén & Muthén, Los Angeles). Descriptive statistics were presented as mean ± standard deviation or [M(Q1, Q3)] for continuous variables and frequency (%) for categorical variables. In longitudinal comparisons, repeated measures ANOVA (assuming sphericity) was used for continuous variables at 3 time points, and Cochran *Q* test was employed for the changes in positive rates. Pearson/Spearman correlation coefficients were calculated between variables. CLPM with autoregressive and theory-driven cross-lagged paths was constructed. Model fit was considered acceptable if χ^2^/df < 3, Comparative Fit Index (CFI) > 0.90, and RMSEA < 0.08. All models utilized full information maximum likelihood to handle missing data. The significance level was set at α = 0.05 (two-tailed).

## 3. Results

### 3.1. General information of the postpartum women

A total of 389 postpartum women were ultimately included in this study. The mean age of the participants was 29.6 ± 4.1 years. Detailed sociodemographic and obstetric characteristics are presented in Table 1.

**Table 1:** General characteristics of the postpartum women.

Characteristic	Category	Number (n)	Percentage (%)
Age (yr)	20–25	58	14.9
	26–30	156	40.1
	>30	175	45.0
Education level	High school or below	62	15.9
	Associate degree	105	27.0
	Bachelor’s degree or above	222	57.1
Monthly family income (RMB)	<10,000	78	20.1
	–20,000	187	48.1
	>20,000	124	31.9
Residence	Urban	312	80.2
	Rural	77	19.8
Delivery mode	Vaginal delivery	211	54.2
	Cesarean section	178	45.8
Newborn sex	Male	202	51.9
	Female	187	48.1

### 3.2. Longitudinal score comparisons of psychological resilience, emotional symptoms and their correlation in postpartum women

**Figure 1.**
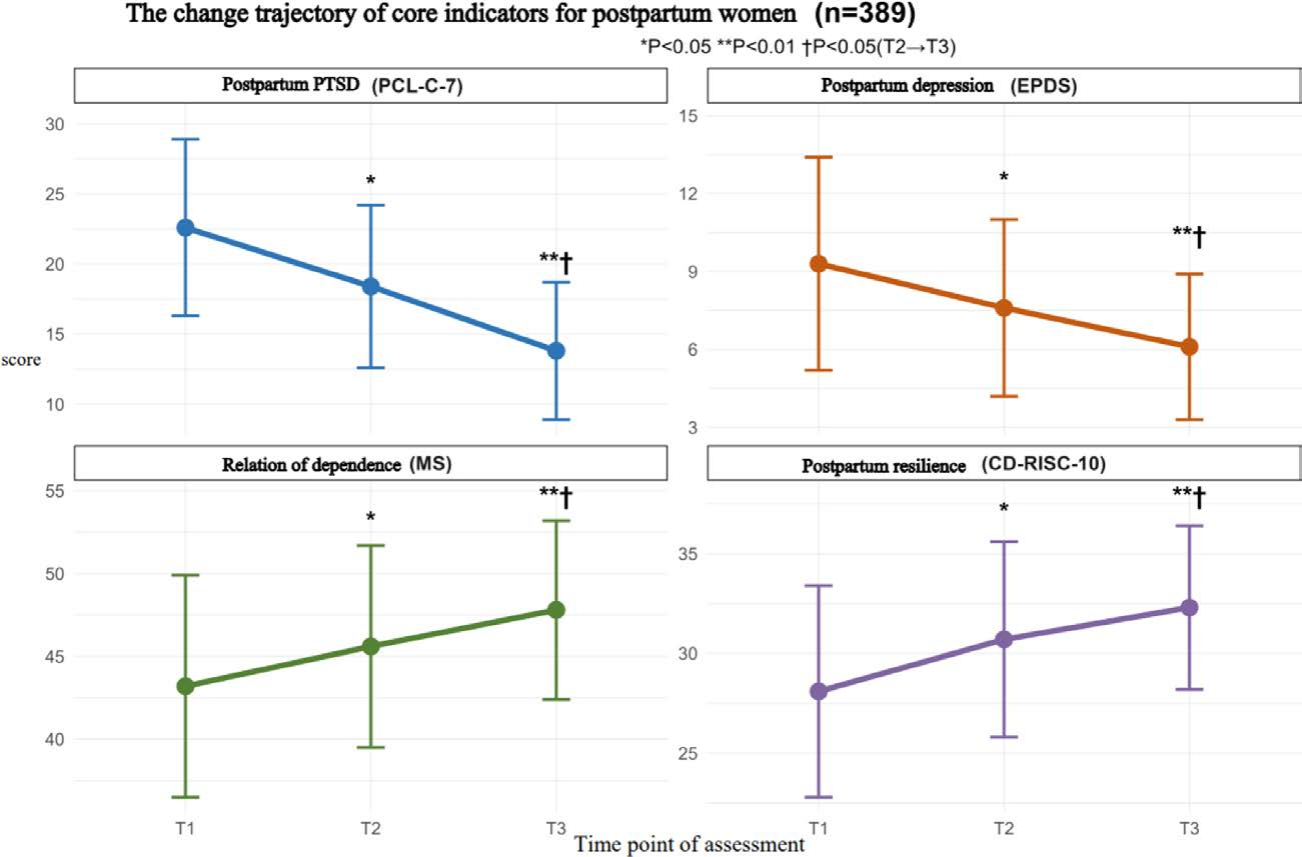
Trajectory of core indicators changes in postpartum women within 3 mo.

The changes in scores for psychological resilience (CD-RISC-10), depression (EPDS), PTSD (PCL-C-7), and interdependence (MS) at 3 time points – T1 (before discharge), T2 (42 days postpartum), and T3 (3 months postpartum) – are presented in Table 2 and Figure 1. A repeated measures ANOVA indicated that the CD-RISC-10 score significantly increased over time (*F* = 24.37, *P* < .001), with the greatest difference observed between T1 and T3 (*P* < .001). The EPDS and PCL-C-7 scores significantly decreased (FEPDS = 18.92, FPCL-C-7 = 16.81, both *P* < .001), with the T3 score being significantly lower than that of T1 (*P* < .01). Additionally, the MS score gradually improved (*F* = 9.85, *P* = .002), with a significant increase from T1 to T3 (*P* = .003).

**Table 2:** Comparison of core variable scores of postpartum women at 3 follow-ups (*x*ˉ±*s*).

Point-in-time	CD-RISC-10 (points)	EPDS (points)	PCL-C-7 (points)	MS (points)
T1	28.1 ± 5.3	9.3 ± 4.1	22.6 ± 6.3	43.2 ± 6.7
T2	30.7 ± 4.9*	7.6 ± 3.4*	18.4 ± 5.8*	45.6 ± 6.1*
T3	32.3 ± 4.1**,***	6.1 ± 2.8**,***	13.8 ± 4.9*,*,*,*,*	47.8 ± 5.4**^,^***
*F*-value	24.37	18.92	16.81	9.85
*P*-value	<.001	<.001	<.001	.002

CD-RISC-10: The total score ranges from 0 to 40, with a higher score indicating better resilience.

EPDS = a score of ≥10 is the critical value for depression risk, PCL-C-7 = a score of ≥38 is the critical value for a positive PTSD diagnosis.

* T2 compared with T1, *P* < .05.

** T3 compared with T1, *P* < .01.

*** T3 compared with T2, *P* < .05.

### 3.3. Results of correlation analysis

The correlation analysis indicates that psychological resilience exhibits a consistent negative correlation with emotional symptoms; furthermore, psychological resilience is positively correlated with the couple’s interdependence, and this correlation intensifies over time. Additionally, depression is strongly positively correlated with PTSD, and the negative correlation between the marital relationship and emotional symptoms becomes increasingly pronounced over time. For further details, refer to Table 3.

**Table 3:** Correlation analysis results of core variables after delivery.

Variate	Correlation coefficient (*r*)		
	T1	T2	T3
CD-RISC-10 and EPDS	−0.62	−0.59	−0.65
CD-RISC-10 and PCL-C-7	−0.58	−0.55	−0.61
CD-RISC-10 and MS	0.49	0.52	0.58
EPDS and PCL-C-7	0.71	0.68	0.63
EPDS and MS	−0.54	−0.57	−0.61
PCL-C-7 and MS	−0.51	−0.53	−0.59

CD-RISC-10 = 10-item Connor–Davidson resilience scale, EPDS = Edinburgh postnatal depression scale, MS = mutuality scale, PCL-C-7 = PTSD Checklist-Civilian version 7, PTSD = post-traumatic stress disorder.

### 3.4. Scale measurement invariance testing

The measurement invariance of the core scales at 3 time points was verified by using multiple-group confirmatory factor analysis. All scales passed the tests of configural invariance and weak invariance (ΔCFI ≤ 0.007, ΔRMSEA ≤ 0.003) as shown in Table 4.

**Table 4:** Results of invariance testing at multiple time points.

Scale	Level of invariance	χ^2^/df	CFI	ΔCFI	RMSEA	ΔRMSEA
CD-RISC-10	Configural invariance	2.15	0.965	–	0.055	–
	Metric invariance	2.27	0.958	0.007	0.058	0.003
EPDS	Configural invariance	2.08	0.961	–	0.053	–
	Metric invariance	2.22	0.954	0.007	0.056	0.003
PCL-C-7	Configural invariance	1.97	0.970	–	0.049	–
	Metric invariance	2.05	0.964	0.006	0.052	0.003
MS	Configural invariance	2.12	0.962	–	0.054	–
	Metric invariance	2.24	0.955	0.007	0.057	0.003

The criterion for judgment: If ΔCFI ≤ 0.010 and ΔRMSEA ≤ 0.015, invariance is considered valid.

CD-RISC-10 = 10-item Connor–Davidson resilience scale, CFI = Comparative Fit Index, EPDS = Edinburgh postnatal depression scale, MS = mutuality scale, PCL-C-7 = PTSD Checklist-Civilian version 7, PTSD = post-traumatic stress disorder.

### 3.5. Results of cross-lagged path analysis

This study employed the CLPM to examine the dynamic interactions between psychological resilience, dyadic coping, PPD, and PTSD across 3 time points. The final model demonstrated excellent fit, with all indices meeting excellent standards: χ^2^/df = 2.35, CFI = 0.96, TLI = 0.94, RMSEA = 0.06, SRMR = 0.04. FIML estimation was used to handle missing data, an effective method for managing data missing at random. The overall missing data rate across the 3 time points was 8.7%. Little’s Missing Completely at Random (MCAR) test results (χ^2^ = 32.15, df = 28, *P* = .27) indicated that the data met the assumption of missing completely at random, suggesting that the use of FIML estimation would not introduce bias. Further analysis revealed no significant association between missingness and baseline demographic characteristics (age, education level, mode of delivery) (*P* > .05).

#### 3.5.1. Dynamic enhancement of protective resources

This study found that both postpartum psychological resilience and dyadic coping exhibited significant temporal stability (autoregressive effects β = 0.50–0.65, *P* < .001), with psychological resilience showing the highest stability (T1 → T2: β = 0.65; T2 → T3: β = 0.62). More importantly, psychological resilience significantly predicted subsequent dyadic coping quality. Specifically, women with higher psychological resilience at discharge showed significantly improved dyadic coping quality at 6 weeks postpartum (β = 0.28, *P* < .001), and this promotive effect persisted until 3 months postpartum (β = 0.26, *P* = .002). This suggests that early interventions targeting psychological resilience can not only enhance individual coping capacity but also indirectly improve the quality of couple interactions.

High-quality dyadic coping played an important protective role in postpartum emotional health. The study found that women with higher relationship quality at 6 weeks postpartum subsequently experienced a significant reduction in depressive symptoms (T1 → T2: β = −0.20, *P* = .008; T2 → T3: β = −0.22, *P* = .003). Simultaneously, psychological resilience directly reduced the risk of depression (T1 → T2: β = −0.25, *P* < .001; T2 → T3: β = −0.23, *P* = .001). This highlights the critical role of partner support in postpartum psychological adaptation, and this protective effect strengthened over the postpartum period, indicating the cumulative benefit of relational resources.

#### 3.5.2. Vicious cycle between symptoms

**Figure 2.**
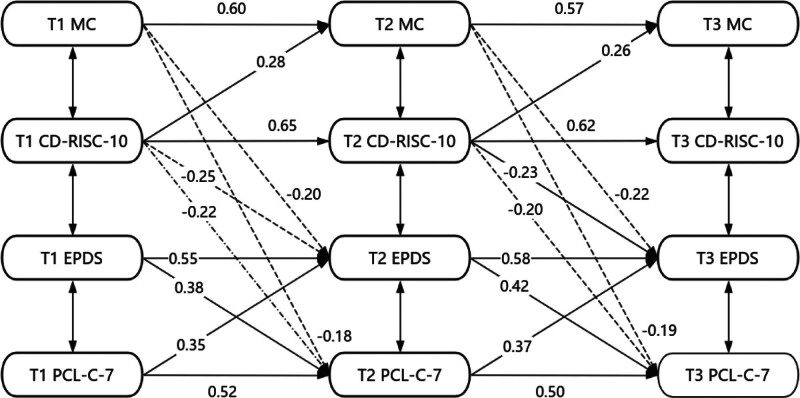
Cross-lagged model of interdependence and psychological symptom clusters in postpartum women.

This study identified a significant bidirectional reinforcement relationship between depressive and PTSD symptoms. Depression exacerbated subsequent PTSD risk (T1 → T2: β = 0.38, *P* < .001; T2 → T3: β = 0.42, *P* < .001), while PTSD also worsened subsequent depression severity (T1 → T2: β = 0.35, *P* < .001; T2 → T3: β = 0.37, *P* < .001). This bidirectional reinforcement effect formed a vicious cycle that maintained symptoms. See Figure 2 for details.

## 4. Discussion

This study systematically explored, for the first time, the dynamic interaction among postpartum women’s psychological resilience, marital interdependence, and the depression-PTSD symptom cluster through 3 waves of longitudinal investigation. Utilizing a cross-lagged design, it revealed the complex mechanisms of key protective and risk factors at various postpartum periods, offering a new perspective for understanding the maintenance path of postpartum mental health.

The scores for psychological resilience exhibited a consistent upward trend at 3 distinct time points. Throughout the process of child-rearing, postpartum women experienced increased self-efficacy as their parenting skills improved, which in turn enhanced their coping abilities.^[20]^ Research indicates that the reorganization of prefrontal cortex function and the recovery of gray matter volume in women are completed 3 months postpartum, offering a neural basis for the enhancement of psychological resilience. Individuals with high psychological resilience are more likely to obtain social support, and this increase in social support further promotes the improvement of psychological resilience, creating a positive feedback loop.^[21,22]^ The score reflecting the interdependence between couples demonstrated a linear improvement trend, and statistical tests revealed a significant main effect of time (*F* = 36.82). The reasons for this improvement are multifaceted: firstly, the deepening of role adaptation and collaboration provided the foundational driving force.^[23]^ The efficiency of the division of labor in parenting improved from T1 to T2, the frequency of negative communication decreased, and the degree of role consensus significantly increased. Secondly, the strengthening of the emotional connection provided crucial support. Biologically, the level of oxytocin increased compared to the baseline 3 months later,^[24]^ and the frequency of intimate behaviors increased. Cognitively, parents’ positive interpretation bias towards co-parenting behaviors was strengthened.^[25]^

The PPD score gradually decreased from 9.3 at T1 to 6.1 at T3, aligning with Ling’s research.^[26]^ This significant downward trend is the result of the combined effect of physiological regulation and psychological adaptation. Physiologically, the rapid stabilization of postpartum hormone levels is the core driving force for the relief of depressive symptoms in the early stage (T1–T2). After delivery, the levels of estrogen and progesterone gradually return to a stable state from a sudden drop, and the fluctuation range of stress hormones such as cortisol decreases. The reconstruction of the neuroendocrine system effectively reduces emotional susceptibility.^[27]^ Moving into the T2 to T3 stage, psychological adaptation mechanisms begin to play a dominant role. As parenting practices deepen, the sense of competence in parenting among new mothers significantly increases, self-efficacy grows, and the ability to interpret the needs of infants and the proficiency in coping skills greatly alleviate the anxiety of “being unable to fulfill the role of a mother.”^[28]^ The postpartum PTSD score decreased from 22.6 at T1 to 13.8 at T3, indicating a rapid trend of relief. This change is the result of the combined effect of trauma memory processing and the strengthening of social support. From the perspective of trauma memory processing, the first 3 months after childbirth is a critical window period for the reconsolidation of trauma memory. As time progresses, the cognitive restructuring of the traumatic delivery event by the parturient is gradually completed.^[29]^ fMRI shows that the activation of the amygdala, responsible for emotional processing, is significantly weakened, resulting in a significant reduction in trauma-related intrusive symptoms and avoidance behaviors. The strengthening of the social support system plays an indispensable role in the relief process. The emotional support from the partner is particularly crucial. For every 1-point increase in the support intensity, the risk of PTSD decreases by 0.21 points.^[30]^ This kind of support not only provides a sense of security but also promotes the expression of traumatic experiences by the parturient, reducing feelings of loneliness and alienation.

The correlational analyses in this study revealed that the relationships between variables exhibited dynamic evolutionary characteristics. These temporal patterns provide important clues for understanding the psychological adaptation process during the postpartum period. First, the positive correlation between psychological resilience and dyadic coping strengthened over time (increasing from *r* = 0.49 at T1 to *r* = 0.58 at T3). This finding may reveal an “upward spiral” effect of resource accumulation.^[31]^ In the early postpartum period, infant needs typically become the absolute center of family attention, while couples’ interaction patterns face restructuring and challenges.^[32]^ Individuals with higher psychological resilience, due to their enhanced emotion regulation abilities and positive coping styles, may be more capable of initiating supportive marital communication and less likely to transform parenting stress into conflicts between partners. These positive interactive behaviors are continuously repeated and reinforced over time, gradually improving the overall atmosphere and quality of the marital relationship. Conversely, high-quality dyadic coping also provides individuals with more emotional support and practical assistance, thereby further nurturing and enhancing their psychological resilience. Thus, personal internal resources and external relational resources may mutually facilitate each other, forming a virtuous cycle of gain that makes the association between them increasingly stronger over time. Second, although the strong positive correlation between depressive and PTSD symptoms persisted, it slightly weakened (decreasing from *r* = 0.71 at T1 to *r* = 0.63 at T3). This subtle change may reflect differentiated developmental pathways of symptoms. In the very early postpartum period (T1), the 2 symptom clusters may share higher common variance, both driven by the intense stress response triggered by the acute event of childbirth, manifesting as a generalized “distress” state.^[33]^ As time progresses into the mid-to-late postpartum period (T3), the specific manifestations of the 2 disorders may become more prominent. For example, depression may more persistently present as low mood, loss of interest, and fatigue, while PTSD may more uniquely manifest as childbirth trauma-related intrusive memories, avoidance, and hypervigilance symptoms. This symptom differentiation process may lead to a slight weakening of their correlation. In summary, these dynamic correlation patterns indicate that postpartum psychological adaptation is not a static outcome but rather a dynamic process full of interactions.

The research results strongly confirm that psychological resilience has a continuous promoting effect on marital relationships. Specifically, it is manifested as the influence of resilience at T1 on interdependence at T2 (β = 0.28), and the influence of psychological resilience at Time 2 (T2) on interdependence at Time 3 (T3) (β = 0.26). This finding provides solid support for the applicability of the resource conservation theory^[34]^ in the postpartum context. Individuals with high resilience are better at mobilizing their own resources to cope with various pressures during the parenting process and enhance the partner relationship through 2 pathways.^[35,36]^ At the cognitive level, it can promote constructive communication and increase the frequency of positive verbal interaction; at the behavioral level, it can enhance parenting collaboration efficiency and improve task allocation efficiency. From a clinical perspective, the early postpartum period (T1) is a critical window for resilience intervention. Therefore, it is recommended to conduct mindfulness-based cognitive restructuring training along with problem-solving skills training. The quality of the marital relationship has a significant negative predictive effect on subsequent emotional symptoms. Specifically, the influence of the relationship at Time 2 (T2) on depression at Time 3 (T3) (β = −0.22), and the influence of the relationship at Time 2 (T2) on PTSD at Time 3 (T3) (β = −0.19). This effect reaches its peak at 42 days postpartum (T2), which is consistent with the expectations of the family stress model.^[37]^ The mechanism mainly reflects the triple buffering effect.^[38,39]^ In terms of emotional buffering, it can provide a sense of security, reduce threat perception, and lower cortisol peaks; in terms of cognitive buffering, it can promote the reevaluation of positive events; in terms of behavioral buffering, it can increase healthy coping behaviors and reasonably increase the frequency of seeking help. In clinical practice, the MS scale should be routinely used to assess the marital relationship during the 42-day postpartum review. For those with an average score of <2.5 on the MS items, partner emotional expression training should be initiated; for those with higher scores, the existing support model should be strengthened, and monthly maintenance sessions should be conducted.

Research has revealed a bidirectional positive feedback loop between depression and PTSD. Examining the pathway from depression to PTSD, there is a significant positive influence from Time 1 to Time 2 (β = 0.38) and from Time 2 to Time 3 (β = 0.42); on the pathway from PTSD to depression, there is also a significant positive effect from Time 2 to Time 3 (β = 0.37). These findings offer support for the symptom network theory,^[40,41]^ particularly as it manifests in the attention bias cycle and the behavioral avoidance cycle. In the attention bias cycle, depression amplifies threat vigilance, thereby increasing the likelihood of traumatic recall; in the behavioral avoidance cycle, PTSD induces social withdrawal, thus diminishing positive experiences. Consequently, a symptom co-control mechanism should be established. Initially, automatically screen for PTSD in individuals with an EPDS score of ≥10; secondly, conduct cognitive behavioral therapy^[42]^ concurrently, such as combining attention training with exposure therapy.

The theoretical contributions of this study are threefold: first, it validates the applicability boundaries of the COR theory during the postpartum period; second, it reveals the central role of marital relationships as a core hub within the family system; and third, it provides longitudinal evidence supporting the network theory of psychopathology. Based on the findings, we propose an empirically supported three-tier intervention strategy: primary intervention should be implemented at T1 (pre-discharge) targeting individuals with low CD-RISC scores through specific techniques such as mindful breathing and cognitive restructuring to enhance psychological resilience and prevent early resource loss; secondary intervention at T2 (6 weeks postpartum) should focus on couples with MS scores <2.5, emphasizing communication training and establishing shared parenting goals to strengthen partner support as a key mediating pathway; tertiary intervention at T3 (3 months postpartum) should target individuals with EPDS ≥ 10 or PCL ≥ 38 scores, adopting comorbidity intervention strategies based on symptom networks, such as cognitive processing therapy simultaneously addressing both depression and PTSD, to break their mutual reinforcement cycle.

This study has several limitations. Although the cross-lagged model provides preliminary inferences about temporal relationships between variables, it cannot completely rule out the influence of unmeasured confounding variables. Future research could consider employing autoregressive cross-lagged models or panel models for more robust causal testing. Additionally, all measurements were based on discrete time points, which cannot capture the continuous dynamic changes in postpartum psychological adaptation. High-frequency ecological momentary assessment may serve as a useful complementary method in future studies. Furthermore, the sample was drawn from a single cultural background, and the generalizability of the conclusions across cultural contexts remains to be verified, particularly in societies with significant differences in family structures and gender roles. Finally, although multiple covariates were controlled, unknown confounding factors may still exist. An honest acknowledgment of these limitations clarifies the applicable boundaries of the study’s conclusions and provides direction for future research. In summary, psychological resilience indirectly alleviates emotional symptoms by enhancing the quality of marital relationships, while depression and PTSD symptoms form a mutually reinforcing cycle over time. The results suggest that resilience training should be initiated early postpartum (T1), marital relationship interventions should be strengthened during the mid-postpartum period (T2), and combined depression-PTSD control strategies should be implemented for individuals at high risk of symptoms. Future research should focus on high-risk populations and conduct cross-cultural validations.

## 5. Conclusion

Research findings suggest that psychological resilience, as a crucial personal asset, not only directly mitigates the risk of depression but also indirectly enhances emotional health by reinforcing the interdependence between spouses. This “resilience-relationship” protective pathway is especially significant during the early postpartum period, offering a key target for preventive interventions. Depression and PTSD create a self-reinforcing negative cycle, with the risk of each condition exacerbating the other increasing over time following childbirth. The early postpartum period represents the optimal window for psychological resilience interventions, while the mid-postpartum period is critical for bolstering the marital relationship. The ideal time for concurrent symptom management is approximately 3 months postpartum. This study has confirmed the protective pathway of “psychological resilience → marital relationship → emotional health” and the risk cycle of “depression-PTSD,” providing a theoretical foundation for phased and precise interventions in postpartum mental health. Future research should aim to deepen the understanding of these mechanisms through interdisciplinary collaboration and to refine the clinical translation process. It is important to note that this study has limitations. It did not include special groups of mothers, such as those who conceived through assisted reproductive technology or those experiencing multiple pregnancies. Therefore, the generalizability of the findings to high-risk groups must be verified with caution. Additionally, the absence of synchronous monitoring of neuroendocrine indicators, such as cortisol and oxytocin, precluded the analysis of the multi-level interaction between biology and behavior. The use of discrete time point measurements may have resulted in the oversight of subtle changes at critical transition points.

## Acknowledgments

We would like to thank the entire medical staff of the Department of Obstetrics, The First People’s Hospital of Nantong, Jiangsu Province.

## Author contributions

**Conceptualization:** Yun Juan Ji, Lili Xue.

**Data curation:** Yun Juan Ji, Lili Xue.

**Formal analysis:** Yun Juan Ji, Lili Xue.

**Investigation:** Liping Chen.

**Methodology:** Liping Chen.

**Project administration:** Yun Juan Ji, Liping Chen.

**Resources:** Yun Juan Ji, Liping Chen.

**Software:** Yun Juan Ji.

**Writing – original draft:** Yun Juan Ji, Lili Xue.

**Writing – review & editing:** Yun Juan Ji, Liping Chen.
